# Cerebrospinal fluid flow dynamics in Huntington's disease evaluated by phase contrast MRI

**DOI:** 10.1111/ejn.14356

**Published:** 2019-02-19

**Authors:** Filipe B. Rodrigues, Lauren M. Byrne, Enrico De Vita, Eileanoir B. Johnson, Nicola Z. Hobbs, John S. Thornton, Rachael I. Scahill, Edward J. Wild

**Affiliations:** ^1^ UCL Huntington's Disease Centre UCL Queen Square Institute of Neurology University College London London UK; ^2^ Neuroradiological Academic Unit UCL Queen Square Institute of Neurology University College London London UK; ^3^ Department of Biomedical Engineering School of Biomedical Engineering and Imaging Sciences King's College London London UK; ^4^ Ixico Plc London UK

**Keywords:** cerebrospinal fluid, humans, Huntington disease, magnetic resonance imaging

## Abstract

Multiple targeted therapeutics for Huntington's disease are now in clinical trials, including intrathecally delivered compounds. Previous research suggests that CSF dynamics may be altered in Huntington's disease, which could be of paramount relevance to intrathecal drug delivery to the brain. To test this hypothesis, we conducted a prospective cross‐sectional study comparing people with early stage Huntington's disease with age‐ and gender‐matched healthy controls. CSF peak velocity, mean velocity and mean flow at the level of the cerebral aqueduct, and sub‐arachnoid space in the upper and lower spine, were quantified using phase contrast MRI. We calculated Spearman's rank correlations, and tested inter‐group differences with Wilcoxon rank‐sum test. Ten people with early Huntington's disease, and 10 controls were included. None of the quantified measures was associated with potential modifiers of CSF dynamics (demographics, osmolality, and brain volumes), or by known modifiers of Huntington's disease (age and *HTT* CAG repeat length); and no significant differences were found between the two studied groups. While external validation is required, the attained results are sufficient to conclude tentatively that a clinically relevant alteration of CSF dynamics – that is, one that would justify dose‐adjustments of intrathecal drugs – is unlikely to exist in Huntington's disease.

**Abbreviations**
ASO: Antisense oligonucleotide
CAG: Cytosine‐adenine‐guanine triplet
CNS: Central nervous system
CSF: Cerebrospinal fluid
CVFT: Categorical Verbal Fluency Test
DBS: Disease Burden Score
FA: Functional Assessment
HTT: Huntingtin
IQR: Interquartile range
ICC: Interclass correlation
IS: Independence Scale
LOI: Level of interest
MIDAS: Medical Image Display Analysis Software
MRI: Magnetic resonance imaging
PBA‐s: Problem Behaviors Assessment Short
PCMRI: Phase contrast magnetic resonance imaging
ROI: Region of interest
SAMPL: Statistical Analyses and Methods in the Published Literature guidelines
SDMT: Symbol Digit Modality Test
SPM12: Statistical Parametric Mapping 12
STROBE: The Strengthening the Reporting of Observational Studies in Epidemiology Statement
T1: First thoracic vertebral body
T8: Eighth thoracic vertebral body
TE: Echo time
TIV: Total intracranial volume
TFC: Total Functional Capacity
TMS: Total Motor Score
TR: Repetition time
UHDRS: Unified Huntington's Disease Rating Scale

## 1 INTRODUCTION

Huntington's disease is a fatal, incurable inherited neurodegenerative disorder. Caused by a CAG (cytosine‐adenine‐guanine)‐triplet repeat expansion in the *HTT* gene, symptoms usually begin in mid‐adulthood, and include motor, cognitive, and psychiatric disturbances (Bates et al., 2015; Rodrigues et al., 2017). Among the most promising therapeutic strategies are antisense oligonucleotides (ASOs), administered intrathecally, intended to diminish production of the causative mutant huntingtin protein (Wild & Tabrizi, 2017).

In the subarachnoid space throughout the neuraxis, cerebrospinal fluid (CSF) has a pulsatile to and fro movement, with local exchanges happening among CSF, interstitial fluid, and blood (Brinker, Stopa, Morrison, & Klinge, 2014). This oscillation can also be used for drug delivery from the spinal intrathecal space to the brain. In Huntington's disease, three experimental therapeutic ASOs are currently being tested by this route (reviewed in Rodrigues & Wild, 2017; Wild & Tabrizi, 2017; Rodrigues & Wild, 2018). It is important to understand whether the delivery of intrathecally administered substances to the brain may be altered by disease‐related variations in CSF flow.

There is evidence that CSF flow dynamics could be altered in Huntington's disease. Large cohort studies have identified clear patterns of brain atrophy (Paulsen et al., 2006; Tabrizi et al., 2013) that might alter CSF dynamics by changing resistance to CSF flow. Furthermore ependymal cilia are morphologically abnormal in post‐mortem Huntington's disease patient brain, and reduced CSF flow has been shown in ex vivo organotypic brain slices from transgenic Huntington's disease mice (Keryer et al., 2011). A case report of atypical performance of spinal anaesthesia in an Huntington's disease patient (Draisci et al., 2012) raises the suspicion that these Huntington's disease‐related changes may together produce clinically relevant alterations of CSF dynamics.

This could have important consequences for the distribution of central nervous system (CNS)‐delivered drugs, meriting consideration in the planning of clinical trial regimens. However, despite this, the dynamics of CSF flow through the brain and spine have never been studied directly in humans with Huntington's disease.

Phase Contrast Magnetic Resonance Imaging (PCMRI) generates signal contrast between flowing and stationary nuclei, such that the signal phase in each image pixel is directly proportional to the magnitude of the local flow velocity, enabling the characterisation of fluid dynamics in vivo (Nitz et al., 1992; O'Donnell, 1985). Using PCMRI gated to the cardiac cycle, it is possible to noninvasively measure the dynamics of moving biofluids, specifically CSF, and hence quantify velocity and flow rates.

## 2 MATERIALS AND METHODS

This study was designed in line with The Strengthening the Reporting of Observational Studies in Epidemiology (STROBE) Statement (von Elm et al., 2007). Statistical reporting is in accordance with the Statistical Analyses and Methods in the Published Literature guidelines (SAMPL; Lang & Altman, 2013).

### 2.1 Study design and participants

We conducted a prospective cross‐sectional pilot study named “Phase1‐HD” involving participants enrolled simultaneously in the Enroll‐HD study (CHDI Foundation, 2012) an international multicentre, prospective registry, and in the HD‐CSF study (Byrne, Rodrigues, Johnson, De Vita, et al., 2018; Byrne, Rodrigues, Johnson, Wijeratne, et al., 2018), a single‐centre prospective cohort based at the UCL Huntington's Disease Centre, London, UK.

The main goal of Phase1‐HD was to establish the methods for and feasibility of applying PCMRI to people with Huntington's disease and to generate exploratory data to inform current practice and design future definitive studies.

Ten healthy volunteers were recruited before study start for scanner optimization, and their data were not used in any of the analyses. All gave written informed consent.

Ten people with early manifest Huntington's disease and 10 healthy controls were recruited through the Huntington's Disease Multidisciplinary Clinic at the National Hospital for Neurology & Neurosurgery in London, United Kingdom, from July 2016 to April 2017. All volunteers had to be 18 year of age or older, and to have no known contraindication to MRI or venepuncture. Early manifest Huntington's disease participants were defined as gene expansion carriers (i.e. 40 or more CAG repeats in the longer *HTT* allele) with unequivocal signs of Huntington's disease (Unified Huntington's Disease Rating Scale [UHDRS] Diagnostic Confidence Level of 4) and a good functional status implying early stage disease (UHDRS Total Functional Capacity [TFC] between 13 and 7). Healthy controls were free from significant comorbidities, and those with a family history of Huntington's disease, had all had a negative test for the Huntington's disease mutation. They were age and gender matched to the Huntington's disease participants.

Exclusion criteria were as follows: inability to tolerate any of the procedures involved, chiefly MRI scanning or phlebotomy; any clinically significant and relevant history that could affect the conduct of the study and evaluation of the data, as ascertained by the investigators, or through detailed medical history and screening assessments; inability or unwillingness to participate in an Enroll‐HD core assessment, or for data obtained from Enroll‐HD to be accessed.

The study was compliant with the principles of the Declaration of Helsinki, and applicable components of the International Conference on Harmonisation (ICH) Good Clinical Practice (GCP). It was approved by the London‐Queen Square ethics committee, and all participants gave written informed consent.

### 2.2 Clinical and laboratory assessments

All participants, excluding the optimization controls, were subject to a detailed clinical evaluation before undergoing the MRI scan, including the Enroll‐HD core assessment, which includes the UHDRS‐99 (Huntington Study Group, 1996; Siesling, van Vugt, Zwinderman, Kieburtz, & Roos, 1998). Overall, the core assessment contains: demographic data; height and weight; alcohol, tobacco and drug history; family history; characteristics of the disease; genetic testing history; past medical history; comorbid conditions; pharmacotherapy, non‐pharmacological therapy, nutritional supplements; the UHDRS TMS; the UHDRS TFC; the UHDRS Functional Assessment (FA); the UHDRS Independence Scale (IS); the Symbol Digit Modality Test (SDMT); the Categorical Verbal Fluency Test (CVFT); the Stroop Color Naming Test; the Stroop Word Reading Test; and the Problem Behaviors Assessment Short (PBA‐s; Craufurd, Thompson, & Snowden, 2001). The disease burden score (DBS) – age × (CAG‐35.5) – was calculated for all people with early manifest Huntington's disease (Paulsen et al., 2006, 2008).

A blood sample (approx. 10 ml) was collected in EDTA tubes (BD, NJ, USA) for osmolality measurement. Analytes’ concentrations were quantified by freezing point depression, and osmolality was computed according to the following formula:

2×(Na+K)+urea+glucose.

### 2.3 Structural brain imaging

T1 and T2‐weighted MRI data were acquired on a 3T scanner, using a protocol designed for this study (Supporting Information: Imaging Methods). From T1‐weighted scans we segmented whole‐brain, ventricles, and total intracranial volume (TIV) with Medical Image Display Analysis Software (MIDAS); and volumes of grey and white matter, and intracranial CSF volumes with Statistical Parametric Mapping 12 (SPM12). T2‐weighted scans were stitched and total CSF volumes were calculated using MIDAS, using a protocol that was validated internally. All segmentations underwent visual quality control to ensure accurate delineation of the regions. No scans failed processing. Brain and ventricle volumes are expressed as a percentage of total intracranial volume, to account for overall head size.

### 2.4 CSF flow and velocities quantification

**Figure 1:**
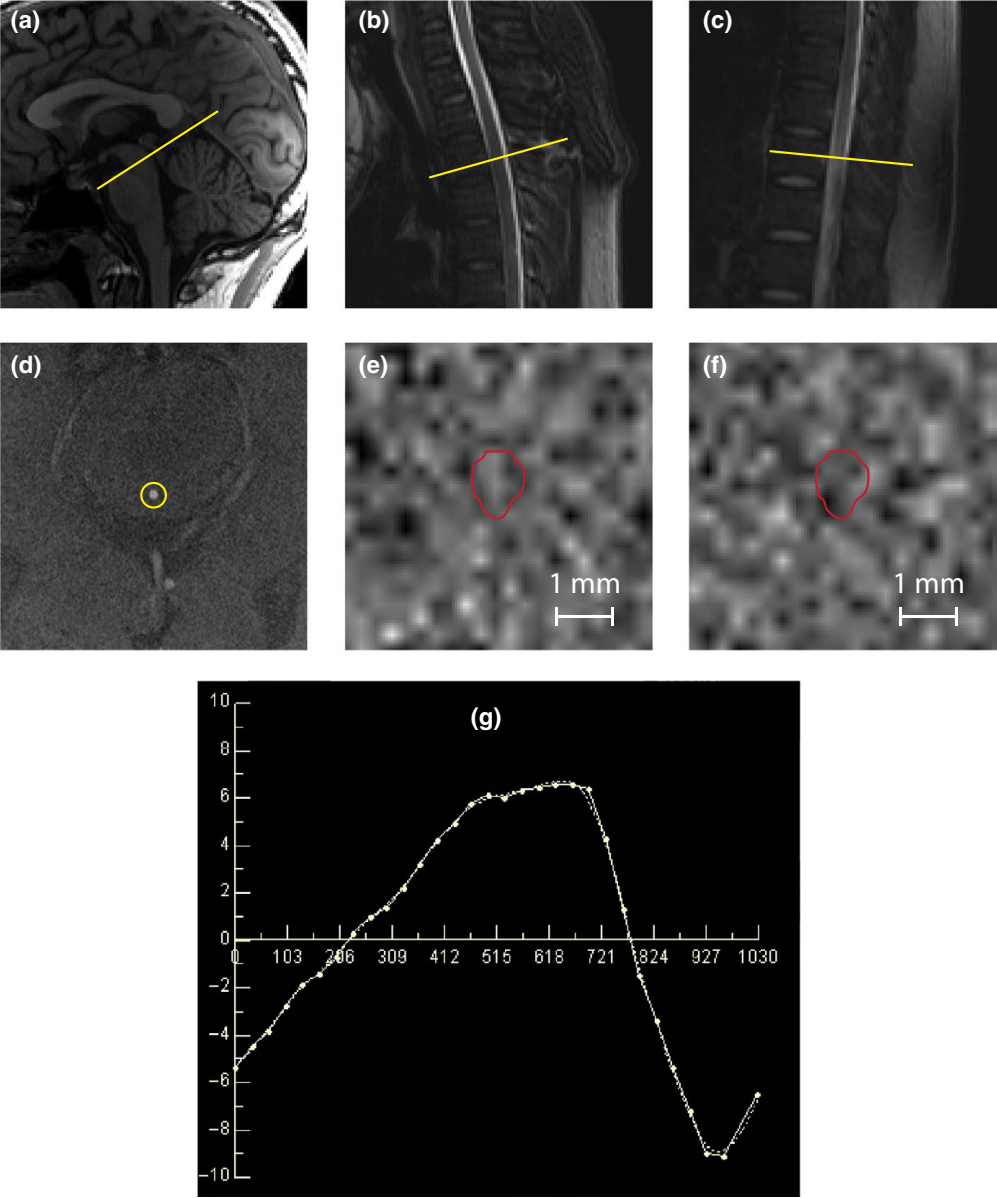
Cerebrospinal fluid dynamics PCMRI analysis pipeline. (a) Representative example of localisation of the cerebral aqueduct on midline sagittal T1‐weighted image; (b) first and (c) eighth thoracic vertebral body localisation on midline sagittal T2‐weighted image. PCMRI analysis was performed on slices perpendicular to the expected direction of flow at the level of interest, selected by an experienced radiographer (yellow lines in a, b and c). (d) Representative axial T2‐weighted MRI at the level of the lamina quadrigemina, showing clearly visualised and delineated cerebral aqueduct (yellow circle). Representative cardiac‐gated PCMRI image at the same level (low intensity representing negative flow velocities (e) and high intensity positive flow (f) at a later phase of the cardiac cycle) together with the manual demarcation of the area of interest (red). (g) Shows an example of a typical normal sinusoidal graph mean of CSF velocity (cm/s) over time (ms) for each phase of the cardiac cycle, in the cerebral aqueduct; data such as these were used to generate the flow and velocity measurements investigated in the study. [Colour figure can be viewed at wileyonlinelibrary.com]

PCMRI was used to quantify CSF velocity and flow at three levels of interest (LOI): the cerebral aqueduct, and the spinal subarachnoid space at the level of the first (T1), and eighth (T8) thoracic vertebral bodies (Figure 1a,b, and c, respectively). Peripheral cardiac gating was performed via an MRI‐compatible pulse oximeter. Oblique‐axial slices for PCMRI were prescribed based on T1‐weighted brain images and T2‐weighted spine images, both perpendicular to the expected direction of flow as assessed by an experienced radiographer. Flow velocity encoding was then performed in the through‐slice direction (perpendicular to the expected direction of flow). The maximum encoding velocity was 20 cm/s. For the cerebral aqueduct, 32 cardiac phases were recorded, with repetition time (TR) = 31 ms, echo time (TE) = 10 ms, field of view 135 × 135 mm (100% phase oversampling), in‐plane spatial resolution 0.53 mm × 0.70 mm and 5 mm slice thickness (acquisition time approx. 6.5 min). For T1 level, 25 cardiac phases were recorded with TR = 31 ms, TE = 10 ms, field of view 135 mm × 135 mm (100% phase oversampling), in‐plane spatial resolution 0.53 mm × 0.70 mm and 5 mm slice thickness (acquisition time approx. 6.5 min). For T8 level, same number of cardiac phases, TE, TR, and slice thickness as for T1, field of view 150 mm × 150 mm (100% phase oversampling), in‐plane spatial resolution 0.59 mm × 0.65 mm (acquisition time approx. 7.6 min). Flow scans underwent quality control at the time of acquisition and prior to processing. This protocol was optimized using the scans of 10 healthy volunteers.

A single set of T8 level PCMRI images from a male manifest HD participant was excluded due to the presence of motion artifact.

Two independent investigators (FBR and RIS), blinded to participant's disease status, analysed all datasets in duplicate. The investigators were instructed to draw manually one region of interest (ROI) per LOI per participant, only selecting pixels with flow during the cardiac cycle, while maximizing the number of pixels included in the analyses. For each ROI, CSF velocity and flow for each cardiac phase were calculated using a built in commercial flow analysis package (Argus Flow, Siemens Healthcare, Erlangen, Germany) as shown in Figure 1; our analyses then used the averages over the complete cardiac cycle. Flow values were calculated in millilitres per minute; mean velocity and peak velocity were calculated in centimetres per second.

### 2.5 Statistical analysis

Statistical analyses were performed using Stata v14.2 (College Station, TX). All analysis were carried out blind to subject group. The threshold for statistical significance for all analyses was *p *<* *0.05.

Inter‐group differences in demographic variables were tested using two‐sample Wilcoxon rank‐sum (Mann–Whitney) test for continuous variables, or Fisher's exact test for categorical variables.

To quantify the agreement between measurements, individual interclass correlations (ICCs) for flow and velocity measurements were calculated between the two raters (FBR and RIS) using a two‐way mixed effect model. Only measures with an ICC over 0.75 were included in the analysis.

For all the analyses below, the velocity and flow values used were a mean of the values generated independently by each investigator.

Non‐parametric Spearman's rank correlation analyses were done to test the effect of age and serum osmolality on CSF flow and velocities in healthy controls, and CAG and DBS in gene expansion carriers. The same was done for brain volumes in healthy controls.

Inter‐group comparisons of CSF flow parameters were performed using a two‐sample Wilcoxon rank‐sum (Mann–Whitney) tests, and as a sensitivity analysis, a *t* test for equal (Student's) or unequal (Welch's) variances according to the variable.

Additional models were constructed to examine the relationship among velocity and flow parameters and clinical data, CAG, DBS, serum osmolality and brain volumes if there was a statistically significant effect on these covariates on the study variables.

Finally, we used the largest magnitude of effect and the most clinically significant variable, as calculated according to the Hedges’ g, to produce sample size computations for future comparisons of two independent means, using an alpha (type I error) of 0.05, and a beta (type II error) of 0.20 and 0.10.

## 3 RESULTS

Twenty participants were recruited, 10 for each group (Table 1). Groups were well balanced in regards to gender, age, ethnicity, and serum osmolality. CSF flow during the cardiac cycle showed a sinusoidal time dependence with cranio‐caudal flow coincident with the first‐phase of the cardiac cycle, followed by caudo‐cranial flow (Figure 1g).

**Table 1:** Participants’ characteristics, and velocity and flow measurements per study group. Peak and mean velocities are shown in cm/s, and mean flows in ml/min

	All	Healthy controls	Manifest HD	*p*‐Value
*N* (%)	20	10 (50%)	10 (50%)	n/a
Males, *n* (%)	10 (50%)	5 (50%)	5 (50%)	1.0000^a^
Age, years, median (IQR)	57 (18.5)	51.5 (13)	60 (8)	0.1120
Caucasian, *n* (%)	20 (100%)	10 (100%)	10 (100%)	1.0000^a^
CAG, median (IQR)	n/a	n/a	42 (3)	n/a
TFC, median (IQR)	13 (2)	13 (0)	11 (2)	0.0002
FA, median (IQR)	25 (2)	25 (0)	23 (3)	0.0002
IS, median (IQR)	100 (20)	100 (0)	80 (15)	0.0002
TMS, median (IQR)	8.5 (22.5)	1.5 (2)	24 (32)	0.0001
SDMT, median (IQR)	40.5 (16)	47.5 (13)	34 (18)	0.0025
CVFT, median (IQR)	23 (7)	24.5 (6)	18.5 (12)	0.0254
SCNT, median (IQR)	65.5 (22.5)	71.5 (11)	50 (14)	0.0022
SWRT, median (IQR)	91 (28.5)	97.5 (19)	69.5 (37)	0.0051
Osmolality, mosmol/kg, median (IQR)	293 (7)	293 (4)	293 (5)	0.4585
CA peak velocity, cm/s, median (IQR)	9.17 (5.40)	7.42 (3.91)	10.72 (4.08)	0.112
CA mean velocity, cm/s, median (IQR)	0.14 (0.18)	0.11 (0.26)	0.15 (0.08)	0.385
CA mean flow, ml/min, median (IQR)	0.29 (0.32)	0.21 (0.36)	0.29 (0.21)	0.570
T1 peak velocity, cm/s, median (IQR)	4.93 (2.28)	5.66 (2.76)	4.93 (1.46)	0.326
T8 peak velocity, cm/s, median (IQR)	5.18 (3.51)	4.99 (2.05)	6.22 (3.28)	0.624

Notes

%: percentage of participants; CA: cerebral aqueduct; CAG: CAG repeat length; CVFT: Categorical Verbal Fluency Test; FA: Unified Huntington's Disease Rating Scale Functional Assessment; IQR: interquartile range; IS: Unified Huntington's Disease Rating Scale Independence Scale; *n*: number of participants; n/a: not applicable; SCNT: Stroop Color Naming Test; SDMT: Symbol Digit Modality Test; SWRT: Stroop Word Reading Test; T1: spinal subarachnoid space at the level of the first thoracic vertebral body; T8: spinal subarachnoid space at the level of the eighth thoracic vertebral body; TFC: Unified Huntington's Disease Rating Scale Total Functional Capacity; TMS: Unified Huntington's Disease Rating Scale Total Motor Score.

a Fisher's exact test, otherwise two‐sample Wilcoxon rank‐sum (Mann–Whitney) test.

Five out of the nine studied measures attained good‐to‐excellent agreement between raters (Supporting Information Table S1) and were further included in the analyses; all others were excluded.

In the healthy controls, we studied the effect of age, gender, serum osmolality, and brain volumes. None of these variables was significantly associated with the study measurements at any level of interest, before or after adjustment for multiple comparisons (Supporting Information Table S2).

**Figure 2:**
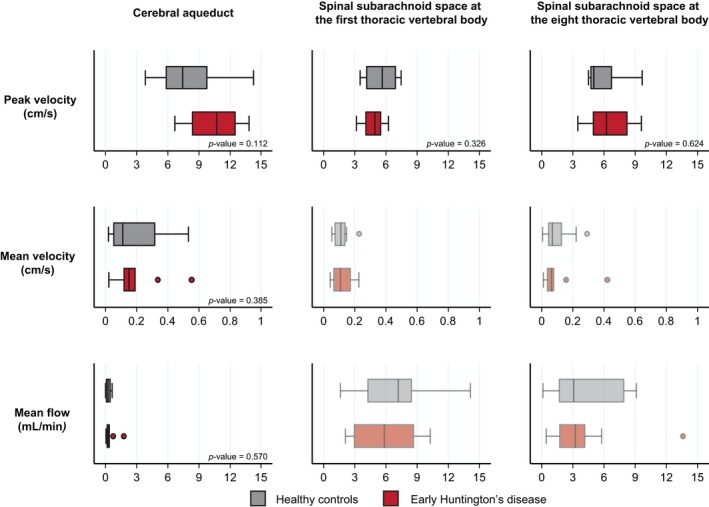
Cerebrospinal fluid flow and velocities do not seem to be different between healthy controls (grey plots) and people with early Huntington's disease (red plots). Faded plots were variables with a sub‐threshold agreement between raters, which were not formally analysed. The boxes extend from the 25th to the 75th percentile with a vertical line for the median (50% percentile). The whiskers extend to the lower and the upper adjacent values (1.5 times the interquartile range plus the 25th or the 75th percentile, respectively). All values outside this range were considered outliers. [Colour figure can be viewed at wileyonlinelibrary.com]

Velocities and flow at each level of interest are shown in Table 1. No significant differences were found between groups for any measure at any level (Figure 2). We then explored whether CSF flow characteristics were related to factors that influence Huntington's disease severity, namely the length of the pathogenic CAG expansion in the *HTT* gene, and its interaction with age. None affected the study measurement consistently at any level of interest, before or after adjustment for multiple comparisons (Supporting Information Table S3).

Finally, we calculated sample sizes for the measure with the largest effect size (peak velocity at the cerebral aqueduct), and of the most clinically meaningful measure for intrathecal bolus injections (peak velocity in the lower spine). Thirty‐seven participants per group would be needed to show a significant difference between two groups with 90% power for the largest effect size and 701 for the most clinically meaningful.

## 4 DISCUSSION

Our results suggest that no clinically significant disease‐related differences in CSF dynamics exist between people with early stage Huntington's disease and healthy controls, at the level of the cerebral aqueduct, upper spine and lower spine. Because this was a small, single‐centre study, our results require external validation, but the attained effect sizes suggest that if such differences do exist, they are likely to be so small that they need not be factored in when designing intrathecal trials in Huntington's disease.

PCMRI has some technical limitations. The strategy used to define the ROIs may limit comparability between studies, and the inherent limit of spatial resolution may introduce error due to partial volume effects. While the adoption of semi‐automated segmentation techniques may improve measurement consistency, to minimise variation we developed a manual delineation protocol to identify regions of interest. This was applied independently by two investigators, blinded to disease group, allowing us to exclude those measures that had a sub‐threshold level of interrater concordance, and the degree of variability in the measures selected for our analysis is in agreement with previous reports.

At the level of the cerebral aqueduct, all three measures had excellent agreement between investigators. The aqueduct is tubular, which favours laminar flow, and has well‐defined margins. In contrast the agreement between investigators was lower in the spinal subarachnoid space. CSF here flows around the spinal cord and is naturally more complex.

Peak velocity was more consistent between measurements based on ROIs defined by different operators, probably because it represents the maximum velocity within the ROI, and thus is essentially independent of the manual anatomical ROI delineation. Mean velocities and flows could be clinically more informative than peak velocities, as they inform on the volume and speed of CSF flow across a region of the sub‐arachnoid space, and not only on an extreme value as the peak velocity does. Unfortunately they are more difficult to determine accurately and precisely, as they depend on the anatomical delineation of the ROI. Our values for flow at the cerebral aqueduct are broadly concordant with previous studies (Huang et al., 2004; Luetmer et al., 2002; Yoshida et al., 2009) and supported by the classic physiological concept that CSF is produced at a rate of 0.3–0.4 ml/min (Brinker et al., 2014).

Overall, our results do not suggest that CSF dynamics are meaningfully altered in early stage Huntington's disease. External validation across disease stages and utilizing a larger sample is needed, but the apparent effect sizes are in keeping with effects that are either absent or of minimal clinical relevance. This is reassuring as targeted molecular therapeutics delivered by bolus intrathecal injection enter large‐scale clinical trials.

## CONFLICT OF INTEREST

EJW has participated in scientific advisory boards with Hoffmann‐La Roche Ltd, Ionis, Shire, GSK and Wave Life Sciences. All honoraria were paid through UCL Consultants Ltd, a wholly owned subsidiary of UCL. His Host Institution, University College London Hospitals NHS Foundation Trust, has received funds as compensation for conducting clinical trials for Ionis Pharmaceuticals, Pfizer and Teva Pharmaceuticals. NZH is an employee of IXICO plc. The other authors declare no competing interests.

## AUTHORS’ CONTRIBUTION

EJW contributed to conceptualization. EJW, JST, EDV, FBR, NZH, and EBJ contributed to methodology. RIS involved in validation. FBR contributed to formal analysis. FBR, EDV, and EBJ involved in investigation. FBR and LMB contributed to resources. FBR involved in curation and writing – original draft. Writing – Review & Editing, LMB, EDV, EBJ, NZH, JST, RIS, and EJW involved in writing – review & editing. EJW contributed to supervision. Project Administration: FBR and LMB involved in project administration. EJW involved in funding acquisition.

## Data Availability

Access to the full dataset used in this work will be granted upon request to the lead author.

## DATA ACCESSIBILITY

Access to the full dataset used in this work will be granted upon request to the lead author.
