# Supplementary material for: Cerebrospinal fluid flow dynamics in Huntington's disease evaluated by phase contrast MRI
Source: Eur J Neurosci. 2019 Feb 19;49(12):1632–9. doi: 10.1111/ejn.14356 (PMC6618296; doi:10.1111/ejn.14356)

**Supplementary Material**

**Supplementary imaging methods**

**Supplementary Table 1** – Consistency of agreement between independent rates.

**Supplementary Table 2** – Exploratory analysis for possible confounders of CSF dynamics**.**

**Supplementary Table 3** – Exploratory analysis for modifiers of Huntington’s disease.

**Supplementary Table 4** – Inter-group comparisons for T1-weighted intracranial CSF volumes and T2- total CSF volumes.

**Supplementary Figure 1** – Representative example after stitching the brain and spinal heavily T2-weighted SPACE sagittal images to quantify total CSF volume.

**Supplementary imaging methods**

T1 and T2-weighted MRI data were acquired on a 3T Siemens PrismaFit scanner with the manufacturer’s 64-channel head/neck coil, using a protocol designed for this study.

T1-weighted images were acquired using a 3D MPRAGE sequence with a TR =2000ms and TE=2.05ms. The protocol had an inversion time of 850ms, flip angle of 8 degrees, matrix size 256x240mm. 256 coronal partitions were collected to cover the entire brain with a slice thickness of 1.0 mm with no gap. Parallel imaging acceleration (R=2) was used (GRAPPA) and 3D distortion correction was applied to all images.

Heavily T2-weighted images were collected to quantify total CSF volume using four SPACE sagittal scans for each participant; a head scan, an upper spine scan, a mid-spine scan and a lower spine scan. The head scan had TR = 2400ms, TE = 762ms, flip angle 100 degrees, turbo factor 161, echo train duration 1055ms, matrix size 256×256mm, and 160 partitions with slice thickness 1mm. The three spine scans has similar parameters, except for 60 partitions (slice oversampling 20%, phase oversampling 50%). Parallel imaging acceleration (R=2) was used (GRAPPA) and 3D distortion correction was applied to all images. The four scans were positioned with a 50mm overlap to ensure that the whole CSF volume was covered. The four scans were stitched on the scanner console using the Siemens Composing toolbox (Supplementary Fig. 1).

Scans underwent visual quality control prior to processing. No T1-weighted scan was excluded due to the presence of significant motion or other artefacts. Two T2-weighted CSF scans were excluded due to failed image stitching.

For T1-weighted scans, bias correction was performed using the N3 procedure(Sled et al., 1998). Volumetric regions of the whole-brain, ventricles and total intracanial volume (TIV) were generated via MIDAS using semi-automated segmentation procedures as previously described (Freeborough et al., 1997, Scahill et al., 2003, Whitwell et al., 2001). SPM12 segmentation (MATLAB version 2012b) was used to quantify the volumes of grey and white matter, and intracranial CSF volumes(Ashburner and Friston, 2005).

After stitching, T2-weighted CSF scans were bias corrected using the N4 algorithm(Tustison et al., 2010). Calculation of total CSF volumes (head and spine) was done via semi-automated segmentation using MIDAS, using a protocol that was validated internally. One rater performed all segmentations, with reproducibility testing completed prior to segmentation to ensure that the rater was segmenting the region with high consistency. Reproducibility testing required the rater to perform delineation on 5 test scans twice, with 5 days between the first and second delineation of each scan (intra-rater reliability). To be validated, the volumes needed to differ less than 5% between the two delineations for each scan. Inter-rater reliability was also tested, with a second rater trained on the procedure, and agreement between the 5 test scans was required to be below 7% difference.

Inter-group comparisons for T1-weighted intracranial CSF volumes and T2-weighted total CSF volumes were performed using a two-sample Wilcoxon rank-sum (Mann-Whitney) tests (Supplementary Table 4).

All segmentations underwent visual quality control to ensure accurate delineation of the regions. No scans failed processing. Brain and ventricle volumes are expressed as a percentage of total intracranial volume, to account for overall head size.

ASHBURNER, J. & FRISTON, K. J. 2005. Unified segmentation. *Neuroimage,* 26**,** 839-51.

FREEBOROUGH, P. A., FOX, N. C. & KITNEY, R. I. 1997. Interactive algorithms for the segmentation and quantitation of 3-D MRI brain scans. *Comput Methods Programs Biomed,* 53**,** 15-25.

SCAHILL, R. I., FROST, C., JENKINS, R., WHITWELL, J. L., ROSSOR, M. N. & FOX, N. C. 2003. A longitudinal study of brain volume changes in normal aging using serial registered magnetic resonance imaging. *Archives of neurology,* 60**,** 989-94.

SLED, J. G., ZIJDENBOS, A. P. & EVANS, A. C. 1998. A nonparametric method for automatic correction of intensity nonuniformity in MRI data. *IEEE transactions on medical imaging,* 17**,** 87-97.

TUSTISON, N. J., AVANTS, B. B., COOK, P. A., ZHENG, Y., EGAN, A., YUSHKEVICH, P. A. & GEE, J. C. 2010. N4ITK: improved N3 bias correction. *IEEE Trans Med Imaging,* 29**,** 1310-20.

WHITWELL, J. L., CRUM, W. R., WATT, H. C. & FOX, N. C. 2001. Normalization of cerebral volumes by use of intracranial volume: implications for longitudinal quantitative MR imaging. *AJNR American journal of neuroradiology,* 22**,** 1483-9.

**Supplementary Table 1 –** Consistency of agreement between independent raters expressed as interclass correlation coefficients. ICC, Interclass correlation coefficient; 95%CI, 95% confidence interval. T1, spinal subarachnoid space at the level of the first thoracic vertebral body; T8, spinal subarachnoid space at the level of the eighth thoracic vertebral body.

| **ICC (95%CI)** | **Level of interest** | | |
| --- | --- | --- | --- |
|  | **Cerebral aqueduct** | **T1** | **T8** |
| **Peak velocity** | 0.97 (0.91 to 0.99)  Excellent | 0.89 (0.75 to 0.96) Good | 0.89 (0.74 to 0.96) Good |
| **Mean velocity** | 0.89 (0.73 to 0.95) Good | 0.71 (0.39 to 0.87) Excluded | 0.40 (-0.05 to 0.72) Excluded |
| **Mean flow** | 0.94 (0.85 to 0.97) Excellent | 0.63 (0.27 to 0.83) Excluded | 0.60 (0.21 to 0.82) Excluded |

**Supplementary Table 2** – Exploratory analysis for possible confounders of CSF dynamics. All co-variates were tested in healthy controls. Shown p-values are not adjusted for multiple comparisons. Using a Bonferroni correction for 5 comparisons, none remains below the threshold for statistical significance (0.05). Associations between continuous variables were computed as Spearman’s correlations. Differences between two groups were tested using a two-sample Wilcoxon rank-sum (Mann-Whitney) test. CA, cerebral aqueduct; CSF, cerebrospinal fluid; T1, spinal subarachnoid space at the level of the first thoracic vertebral body; T8, spinal subarachnoid space at the level of the eighth thoracic vertebral body.

| Variables | **Age** | | **Gender** | **Osmolality** | | **Total brain volume** | | **Ventricle volume** | | **Grey matter volume** | | **White matter volume** | | **Intracranial CSF volume** | | **Total CSF volume** | |
| --- | --- | --- | --- | --- | --- | --- | --- | --- | --- | --- | --- | --- | --- | --- | --- | --- | --- |
|  | rho | p-value | p-value | rho | p-value | rho | p-value | rho | p-value | rho | p-value | rho | p-value | rho | p-value | rho | p-value |
| **CA peak velocity** cm/s | 0.455 | 0.187 | 0.028 | -0.234 | 0.516 | -0.433 | 0.244 | 0.133 | 0.732 | -0.767 | 0.016 | -0.033 | 0.932 | 0.600 | 0.088 | 0.467 | 0.205 |
| **CA mean velocity** cm/s | 0.115 | 0.751 | 0.917 | 0.345 | 0.329 | -0.117 | 0.765 | 0.067 | 0.865 | -0.150 | 0.700 | 0.017 | 0.966 | -0.067 | 0.865 | 0.183 | 0.637 |
| **CA mean flow** mL/min | 0.552 | 0.098 | 0.251 | 0.203 | 0.574 | -0.333 | 0.381 | 0.067 | 0.865 | -0.450 | 0.224 | -0.133 | 0.732 | 0.217 | 0.576 | 0.467 | 0.205 |
| **T1 peak velocity** cm/s | -0.382 | 0.276 | 0.028 | 0.111 | 0.761 | 0.300 | 0.433 | -0.283 | 0.460 | 0.583 | 0.099 | 0.033 | 0.932 | -0.517 | 0.154 | -0.700 | 0.036 |
| **T8 peak velocity** cm/s | -0.182 | 0.614 | 0.917 | -0.055 | 0.879 | 0.300 | 0.433 | -0.483 | 0.188 | -0.083 | 0.831 | 0.417 | 0.265 | -0.217 | 0.576 | -0.417 | 0.265 |

**Supplementary Table 3** – Exploratory analysis for modifiers of Huntington’s disease. All co-variates were tested in people with early manifest HD. Shown p-values are not adjusted for multiple comparisons. Using a Bonferroni correction for 5 comparisons, none remains below the threshold for statistical significance (0.05). Associations between continuous variables were computed as Spearman’s correlations. CA, cerebral aqueduct; CAG, CAG-repeat length; CSF, cerebrospinal fluid; DBS, Disease Burden Score; T1, spinal subarachnoid space at the level of the first thoracic vertebral body; T8, spinal subarachnoid space at the level of the eighth thoracic vertebral body.

| Variables | **CAG** | | **DBS** | |
| --- | --- | --- | --- | --- |
|  |  |  |  |  |
|  | rho | p-value | rho | p-value |
| **CA peak velocity** cm/s | 0.171 | 0.636 | 0.200 | 0.580 |
| **CA mean velocity** cm/s | -0.294 | 0.410 | -0.200 | 0.580 |
| **CA mean flow** mL/min | -0.377 | 0.282 | -0.286 | 0.424 |
| **T1 peak velocity** cm/s | -0.184 | 0.612 | -0.055 | 0.881 |
| **T8 peak velocity** cm/s | 0.084 | 0.830 | 0.083 | 0.831 |

**Supplementary Table 4** – Inter-group comparisons for T1-weighted intracranial CSF volumes and T2- total CSF volumes. Statistical inference was performed using a two-sample Wilcoxon rank-sum (Mann-Whitney) test.

| Variables  median (IQR) | **All** | **Healthy controls** | **Manifest HD** | **p-value** |
| --- | --- | --- | --- | --- |
| **T1 intracranial CSF volume,** mL | 41.96 (21.97) | 28.85 (15.53) | 50.64 (14.29) | 0.0006 |
| **T2 total CSF volume,** mL | 338.16 (195.17) | 255.84 (127.76) | 380.46 (145.72) | 0.0071 |

**Supplementary Figure 1** – Representative example after stitching the brain and spinal heavily T2-weighted SPACE sagittal images to quantify total CSF volume.


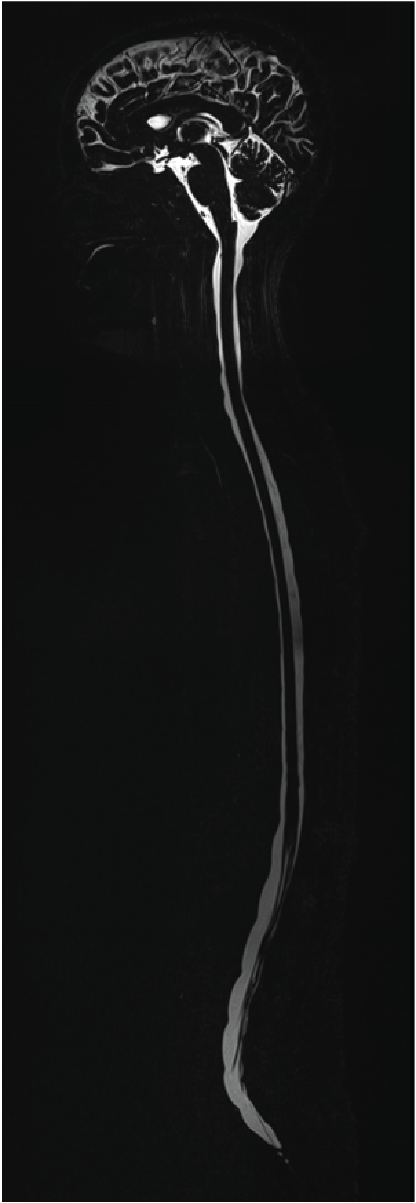

Supplement: Supplementary file 2 [file EJN-49-1632-s002.docx]
