# Supplementary material for: Cerebrospinal fluid flow dynamics in Huntington's disease evaluated by phase contrast MRI
Source: Eur J Neurosci. 2019 Feb 19;49(12):1632–9. doi: 10.1111/ejn.14356 (PMC6618296; doi:10.1111/ejn.14356)
Supplement: Supplementary file 1 [file EJN-49-1632-s001.docx]

| Cerebrospinal fluid flow dynamics in Huntington's disease evaluated by phase contrast MRI | |
| --- | --- |
|  | Rodrigues, Filipe; Byrne, Lauren; De Vita, Enrico; Johnson, Eileanoir; Hobbs, Nicola;  Thornton, John; Scahill, Rachael; Wild, Edward |

_____________________________________________________________________________________

| Review timeline: | Submission date: 30/10/18  Editorial Decision: Minor revision  Revision Received: 11/01/19  Accepted: 22/01/19 |  |
| --- | --- | --- |
|  |  |  |

Editor: Yoland Smith

Reviewer 1: Jaime Hatcher-Martin

Reviewer 2: Anonymous

_____________________________________________________________________________________

1st Editorial Decision	14/12/18

_____________________________________________________________________________________

Cerebrospinal fluid flow dynamics in Huntington's disease evaluated by phase contrast MRI 

Dear Dr. Rodrigues, 

Your manuscript has been reviewed by external reviewers as well as by the Section Editor,  Dr. Yoland Smith, and ourselves.  We are pleased to say that we expect your paper to be acceptable for publication in EJN after some minor revisions. 

We concur with the more positive reviewer that the information provided in this short communication is important and must be made available to the scientific and clinical community in the HD field. Please, address each of the minor concerns raised in this review in a revised version of your manuscript. Although we agree with the more critical reviewer that a larger sample of cases would be better, it is clear based on data presented in the study that a N=10 was sufficient to make meaningful and statistically-relevant conclusions. However, we ask you to address the other points raised by this reviewer and to bring up the issue of a small sample size as a potential limitation that must be taken into consideration when one extrapolates these findings to the large HD population. Editorial  re-review only of the revised manuscript will be necessary. 

Please also address the following points in your revised version. 
- Please reformat the paper to adhere to EJN style i.e. full text file that includes the figure legends and tables at the end (but not figures), separate figure files and the graphic abstract and text as separate files. 
- Check references for journal names e.g. Yoshida et al.   

When revising the manuscript, please embolden or underline major changes to the text so they are easily identifiable and DO NOT leave 'track change' formatting marks in your paper.  Please ensure that you provide a text and a figure file for the Graphical Abstract (as detailed in the instructions below).  When carrying out your revisions please refer to the checklist below and visit the EJN author guidelines at www.ejneuroscience.org 

When finalized, please upload your complete revised manuscript onto the website, as a Word file (.doc, or .docx).  Please also ensure that a complete set of tables and figures is included as separate files, even if these have not changed from the originals.  At this stage it is necessary to provide high resolution figures.  Please see important instructions below. 

Please go into https://mc.manuscriptcentral.com/ejn - Author Centre - manuscripts with decisions where you will find a 'create a revision' link under 'actions'. We ask that you please indicate the way in which you have responded to the points raised by the Editors and Reviewers in a letter. Please upload this response letter as a separate Word (.doc or PDF) file using the file designation "Authors' Response to Reviewers" when uploading your manuscript files.  Please DO NOT submit your revised manuscript as a new one.  Also, please note that only the  Author who submitted the original version of the manuscript should submit a revised version. 

If you are able to respond fully to the points raised, we would be pleased to receive a revision of your paper within 30 days. 

Thank you for submitting your work to EJN. 

Best wishes, 

Paul Bolam & John Foxe 
co-Editors in Chief, EJN 


Reviews: 

Reviewer: 1 

Comments to the Author 
Overall, this is an important paper that is submitted at a very timely moment in HD history.  As more therapies involved intrathecal delivery, it is important to understand flow dynamics, not only in HD, but also in "healthy controls" as future treatments may target individuals without obvious brain atrophy, etc. 

I suggest a few minor comments/revisions that should not affect acceptance of the manuscript: 
- Make sure all abbreviations are defined in the main body of the manuscript. 
- Would recommend commenting on the trend of increased peak velocity of flow in HD even though it did not reach significance. If this is due to an outlier, explain this. 
- Would recommend making a statement as to WHY inter-rater reliability on some measures was poor.  Is this true of these measures in other disease states?  Is this because of inadequate rater training or because it is not an adequate measure?   
- Figure 1: Recommend placing the reference letters for figure (E and F) adjacent to their respective negative and positive flow descriptions for clarity (i.e. not at the beginning of the sentence) 
- Section 2.5 Statistical analyses.  Clarify statement, "The same was done for brain volumes in healthy controls."  Was this not done for brain volume in HD? 
- In discussion - add a short statement explaining WHY mean velocities and flows could be more informative and why they are more difficult to measure. 
- Consider adding a statement or qualifying current statement in Discussion that CSF dynamics are not meaningfully altered in EARLY MANIFEST HD.  Would be interesting to do same analyses in middle (or even late) stage HD.  I did not see this mentioned anywhere in manuscript.   

Reviewer: 2 

Comments to the Author 
The authors used PCMRI to examine the CSF dynamics of human brains with/without HD. this study has significant potential in clinical study relevant to thtrathecal drug delivery to the brain of HD patients.  the hypothesis, experimental design and data analysis are appropriate. however, the results  may be biased due to limited spatial resolution of PCMRI images and small sample size.  Increasing sample size substantially is strong suggested. 

there are some other comments: 
1) Figure 1. scale bars for Figure E and F should be shown.  captions are missing for x and y axis of Fig G. 


2) how many pixels  are included in each LOI (Figure 1F) ? How the PCMRI images were interpolated? 

3) in method section, the full names of the software MIDAS and SPM12 should be given and cited. 

also, TR, TE, HD, LOI, and SPACE are not seen in Abbreviations. 

------------------------ ____________________________________________________________________________________

Authors’ Response

_____________________________________________________________________________________

**EJN-2018-10-25980 – Replies to the comments**

**Editorial Comments:**

Please also address the following points in your revised version. Please reformat the paper to adhere to EJN style i.e. full text file that includes the figure legends and tables at the end (but not figures), separate figure files and the graphic abstract and text as separate files.

**REPLY: The manuscript has been formatted as suggested.**

Check references for journal names e.g. Yoshida et al.

**REPLY: The manuscript has been formatted as suggested.**

**Reviewer: 1**

Overall, this is an important paper that is submitted at a very timely moment in HD history. As more therapies involved intrathecal delivery, it is important to understand flow dynamics, not only in HD, but also in "healthy controls" as future treatments may target individuals without obvious brain atrophy, etc.

**REPLY: We thank the reviewer for their comments.**

I suggest a few minor comments/revisions that should not affect acceptance of the manuscript:

- Make sure all abbreviations are defined in the main body of the manuscript.

**REPLY: We thank the reviewer for this comments. As suggested we added all abbreviations were defined in the main body of the manuscript and in the Abbreviations section.**

- Would recommend commenting on the trend of increased peak velocity of flow in HD even though it did not reach significance. If this is due to an outlier, explain this.

**REPLY: We would like to acknowledge the reviewer for their comment. Our peak flow variables don’t have outliers in any of the levels of interest (Figure 2, first row). The definition of outlier used is a value over/below the upper/lower adjacent value (the median plus or minus 1.5 times the interquartile range [Tukey 1977]), respectively. The results of the statistical tests we defined *a priori* are above the predetermined threshold for statistical significance (i.e. 0.122, 0.326, 0.624), which means that the probability of finding the observed values or more extreme ones, given that the null hypothesis is true (in this case H0: median1 – median2 = 0), is likely (12%, 32% and 62%). Also, in our dataset the direction of the effect is not consistent across levels of interest, and there is an uncorrectable multiplicity bias due to testing multiple hypothesis.**

**We understand it could be relevant to discuss a p-value hovering around the predetermined lever of significance in a pilot/feasibility study, but given the reasons above, in our particular dataset we feel that doing so is best avoided.**

- Would recommend making a statement as to WHY inter-rater reliability on some measures was poor. Is this true of these measures in other disease states? Is this because of inadequate rater training or because it is not an adequate measure?

**REPLY: Thank you for this comment and suggestion. We were very strict in the criteria used to discard measures form the analysis (ICC=>0.75, meaning a high level of inter-rater agreement). The reason why peak velocity is not affected is because it picks up the quickest-flowing pixel in the region of interest, regardless of the demarcation of the region of interest. Usually the quickest-flowing pixels are the central ones and not the ones on the periphery. Mean velocity and flow depend on all the pixels, and hence why they are more affected by the manual demarcation of the region of interest. In summary, the mean velocity and mean flow are always more affected by manual demarcation than peak velocities – and this is a characteristic of the method. Regarding what is observed other studies, although not numerically reported, several authors state that the inter-rater agreement is good for the aqueduct and sub-optimal for the spinal subarachnoid space elsewhere.**

**The text below has been altered accordingly in the manuscript:**

**“At the level of the cerebral aqueduct, all three measures had excellent agreement between investigators. The aqueduct is tubular, which favours laminar flow, and has well-defined margins. In contrast the agreement between investigators was lower in the spinal subarachnoid space. CSF here flows around the spinal cord and is naturally more complex.** **Peak velocity was more consistent between measurements based on ROIs defined by different operators, probably because it represents the maximum velocity within the ROI, and thus is essentially independent of the manual anatomical ROI delineation. Mean velocities and flows could be clinically more informative than peak velocities, as they inform on the volume and speed of CSF flow across a region of the sub-arachnoid space, and not only on an extreme value as the peak velocity does. Unfortunately they are more difficult to determine accurately and precisely, as they depend on the anatomical delineation of the ROI.”**

- Figure 1: Recommend placing the reference letters for figure (E and F) adjacent to their respective negative and positive flow descriptions for clarity (i.e. not at the beginning of the sentence)

**REPLY: Thank you for the suggestion. It has been corrected as suggested. The text below has been altered accordingly in the manuscript:**

***“(A) Representative example of localisation of the cerebral aqueduct on midline sagittal T1-weighted image; (B) first and (C) eighth thoracic vertebral body localisation on midline sagittal T2-weighted image. PCMRI analysis was performed on slices perpendicular to the expected direction of flow at the level of interest, selected by an experienced radiographer (yellow lines in A, B and C). (D) Representative axial T2-weighted MRI at the level of the lamina quadrigemina, showing clearly visualised and delineated cerebral aqueduct (yellow circle). Representative cardiac-gated PCMRI image at the same level (low intensity representing negative flow velocities (E) and high intensity positive flow (F) at a later phase of the cardiac cycle) together with the manual demarcation of the area of interest (red). (G) Shows an example of a typical normal sinusoidal graph mean of CSF velocity (cm/s) over time (ms) for each phase of the cardiac cycle, in the cerebral aqueduct; data such as these were used to generate the flow and velocity measurements investigated in the study.”***

- Section 2.5 Statistical analyses. Clarify statement, "The same was done for brain volumes in healthy controls." Was this not done for brain volume in HD?

**REPLY: The reviewer’s statement is correct. We did not run a correlation analysis between brain volumes and CSF flow measures in HD. Our intent was to test the effect of brain volumes, independently of the effect of the disease, in our outcome measures, and this is why HD cases were excluded from the analysis. The text below has been altered accordingly in the manuscript:**

***“Non-parametric Spearman’s rank correlation analyses were done to test the effect of age, serum osmolality, and brain volumes on CSF flow and velocities in healthy controls, as the former were hypothesized to have an effect independent from the disease effect on the latter. Because CAG and DBS are known predictors of the natural history of HD, these were tested solely in gene expansion carriers.”***

- In discussion - add a short statement explaining WHY mean velocities and flows could be more informative and why they are more difficult to measure.

**REPLY: We thank the review for their suggestion. The following statement was added to the discussion:**

**“Mean velocities and flows could be clinically more informative than peak velocities, as they inform on the volume and speed of CSF flow across a region of the sub-arachnoid space, and not only on an extreme value as the peak velocity does. Unfortunately they are more difficult to determine accurately and precisely, as they depend on the anatomical delineation of the ROI”.**

- Consider adding a statement or qualifying current statement in Discussion that CSF dynamics are not meaningfully altered in EARLY MANIFEST HD. Would be interesting to do same analyses in middle (or even late) stage HD. I did not see this mentioned anywhere in manuscript.

**REPLY: We thank the review for their comment and suggestion. The following statements were altered in line with the reviewers point:**

***“Our results suggest that no clinically-significant disease-related differences in CSF dynamics exist between people with early stage Huntington's disease and healthy controls, at the level of the cerebral aqueduct, upper spine and lower spine”* and *“Overall, our results do not suggest that CSF dynamics are meaningfully altered in early stage Huntington's disease. External validation across disease stages and utilizing a larger sample is needed, but the apparent effect sizes are in keeping with effects that are either absent or of minimal clinical relevance”.***

Reviewer: 2

Comments to the Author

The authors used PCMRI to examine the CSF dynamics of human brains with/without HD. this study has significant potential in clinical study relevant to thtrathecal drug delivery to the brain of HD patients. the hypothesis, experimental design and data analysis are appropriate. however, the results may be biased due to limited spatial resolution of PCMRI images and small sample size. Increasing sample size substantially is strong suggested.

**REPLY: We would like to acknowledge the reviewers points. In the discussion now we emphasize the limitations of the PCMRI and of the study design suggesting that external validation in a larger sample would be needed to draw more conclusive findings. The text below has been altered accordingly in the manuscript:**

***“PCMRI has some technical limitations. The strategy used to define the ROIs may limit comparability between studies, and the inherent limit of spatial resolution may introduce error due to partial volume effects. While the adoption of semi-automated segmentation techniques may improve measurement consistency, to minimise variation we developed a manual delineation protocol to identify regions of interest. This was applied independently by 2 investigators, blinded to disease group, allowing us to exclude those measures that had a sub-threshold level of interrater concordance, and the degree of variability in the measures selected for our analysis is in agreement with previous reports. At the level of the cerebral aqueduct, all three measures had excellent agreement between investigators. The aqueduct is tubular, which favours laminar flow, and has well-defined margins. In contrast the agreement between investigators was lower in the spinal subarachnoid space. CSF here flows around the spinal cord and is naturally more complex. Peak velocity was more consistent between measurements based on ROIs defined by different operators, probably because it represents the maximum velocity within the ROI, and thus is essentially independent of the manual anatomical ROI delineation. Mean velocities and flows could be clinically more informative than peak velocities, as they inform on the volume and speed of CSF flow across a region of the sub-arachnoid space, and not only on an extreme value as the peak velocity does. Unfortunately they are more difficult to determine accurately and precisely, as they depend on the anatomical delineation of the ROI.”* and *“Overall, our results do not suggest that CSF dynamics are meaningfully altered in early stage Huntington's disease. External validation across disease stages and utilizing a larger sample is needed, but the apparent effect sizes are in keeping with effects that are either absent or of minimal clinical relevance.”.***

there are some other comments:

1) Figure 1. scale bars for Figure E and F should be shown. captions are missing for x and y axis of Fig G.

**REPLY: We have changed figure 1 accordingly. The following was added to the figure legend: *“Shows an example of a typical normal sinusoidal graph mean of CSF velocity (cm/s) over time (ms) for each phase of the cardiac cycle, in the cerebral aqueduct; data such as these were used to generate the flow and velocity measurements investigated in the study”.***

2) how many pixels are included in each LOI (Figure 1F) ? How the PCMRI images were interpolated?

**REPLY: The in-plane resolution of the acquisition at the level of the cerebral aqueduct (Figure 1F) was 80mm/256=0.3125mm. The mean diameter of the cerebral aqueduct in our acquisitions was 4.47mm^2 (SD 6.38). As so, on average each aqueduct will have proximately 14 pixels. The interpolation was done with the Siemens Argos Flow software, and the ROI delimitation is was jagged to the pixels. This information was added to the manuscript:**

**“For the cerebral aqueduct, 32 cardiac phases were recorded, with repetition time (TR) = 31ms, echo time (TE) = 10ms, field of view 135 x 135mm (100% phase oversampling), in-plane spatial resolution 0.53mm x 0.70mm and 5mm slice thickness (acquisition time approx. 6.5 min, average 14 pixels).”**

3) in method section, the full names of the software MIDAS and SPM12 should be given and cited. also, TR, TE, HD, LOI, and SPACE are not seen in Abbreviations.

**REPLY: We thank the reviewer for this comments. We made sure all abbreviations were defined in the main body of the manuscript.**
